# Influence of socio-economic and lifestyle factors on overweight and nutrition-related diseases among Tunisian migrants versus non-migrant Tunisians and French

**DOI:** 10.1186/1471-2458-7-265

**Published:** 2007-09-25

**Authors:** Caroline Méjean, Pierre Traissac, Sabrina Eymard-Duvernay, Jalila El Ati, Francis Delpeuch, Bernard Maire

**Affiliations:** 1 Nutrition Unit R106 (WHO collaborating centre for nutrition), Institut de Recherche pour le Développement (IRD), BP 64501, 34394 Montpellier, France; 2 Doctoral School 393 'Public health: epidemiology and biomedical information sciences', Université Pierre et Marie Curie, 75006 Paris, France; 3 Institut National de Nutrition et Technologie Alimentaire (INNTA), 1006 Tunis, Tunisia

## Abstract

**Background:**

Migrant studies in France revealed that Mediterranean migrant men have lower mortality and morbidity than local-born populations for non-communicable diseases (NCDs). We studied overweight and NCDs among Tunisian migrants compared to the population of the host country and to the population of their country of origin. We also studied the potential influence of socio-economic and lifestyle factors on differential health status.

**Methods:**

A retrospective cohort study was conducted to compare Tunisian migrant men with two non-migrant male groups: local-born French and Tunisians living in Tunisia, using frequency matching. We performed quota sampling (n = 147) based on age and place of residence. We used embedded logistic regression models to test socio-economic and lifestyle factors as potential mediators for the effect of migration on overweight, hypertension and reported morbidity (hypercholesterolemia, type-2 diabetes, cardiovascular diseases (CVD)).

**Results:**

Migrants were less overweight than French (OR = 0.53 [0.33–0.84]) and had less diabetes and CVD than Tunisians (0.18 [0.06–0.54] and 0.25 [0.07–0.88]). Prevalence of hypertension (grade-1 and -2) and prevalence of hypercholesterolemia were significantly lower among migrants than among French (respectively 0.06 [0.03–0.14]; 0.04 [0.01–0.15]; 0.11 [0.04–0.34]) and Tunisians (respectively OR = 0.07 [0.03–0.18]; OR = 0.06 [0.02–0.20]; OR = 0.23 [0.08–0.63]).

The effect of migration on overweight was mediated by alcohol consumption. Healthcare utilisation, smoking and physical activity were mediators for the effect of migration on diabetes. The effect of migration on CVD was mediated by healthcare utilisation and energy intake. No obvious mediating effect was found for hypertension and hypercholesterolemia.

**Conclusion:**

Our study clearly shows that lifestyle (smoking) and cultural background (alcohol) are involved in the observed protective effect of migration.

## Background

For several decades, massive migration has taken place from southern countries to industrialised countries where migrants usually experience radical changes in lifestyle. Besides their importance for public health, studies of migrants' health also provide good models to study the importance of the environment of populations for health [1]. Indeed, such studies have been widely conducted in research on cancer epidemiology, in order to disentangle genetic factors from the influence of the environment [2].

In most host countries, migrants belong to underprivileged social backgrounds, and consequently have higher rates of mortality and morbidity than the native population [3-5]. However, in 1986, an epidemiologic paradox was revealed in the USA: compared with the non-Hispanic 'white' population, the Hispanic population had lower death rates for cancer, cardiovascular diseases, and all-cause mortality [6]. This paradox is currently theorized in a wider geographical and cultural context [7-10] and several hypotheses have been proposed to elucidate this effect [11], which could be related to the "healthy migrant effect", i.e. the selection, at entry, of applicants for immigration who are healthier than their average compatriots [12]. Another explanation could be the "salmon bias", theory which supposes that migrants probably migrate back to their home country after the retirement or when they are seriously ill. Powles [13] also proposed an attractive hypothesis: "the best of both worlds"; the continuation, even the preservation, of traditional behaviours favourable to health, more important family support, and a better access to a health care system could protect migrants, and in particular from non-communicable diseases (NCDs).

Although France has long been a country of immigration, few studies have been made on migrant health and particularly on the impact of migration on diet and lifestyle related diseases. However, the studies that are available also showed a paradox among Mediterranean migrant men in France, i.e. that migration could have a protective effect on mortality and morbidity linked to NCDs compared with the local-born population [14-16]. Again, although several hypotheses have been put forward, the origin of this paradox is still unknown [17].

The present work studied the effect of migration on overweight and morbidity linked to NCDs among Tunisian migrants in the south of France and the potential influence of socio-economic and individual lifestyle factors. Like the other southern Mediterranean countries, Tunisia is currently undergoing an epidemiological transition [18] where obesity and the attendant risks of NCDs are a growing public health problem [19,20]. We compared a group of Tunisian migrants first with a local-born French population living in the same environment and second with a non-migrant Tunisian population. The main question addressed was whether Tunisian migrants retain traditional healthy behaviours that could explain the protective effect of migration.

## Methods

### Design and sampling

The study focused on Tunisian migrant men living in Languedoc-Roussillon, a French Mediterranean region which is historically a region of immigration. A retrospective cohort study compared Tunisian migrant men age ≥ 18 y ("migrants") and two non-migrant male groups: French born in France ("French") and Tunisians living in Tunisia ("Tunisians"). Exposed subjects (migrants) were defined as individuals who were natives of Tunisia who had been residing in France for more than one year at the time of the survey. As French law does not allow access to nominative files with ethnic status, random sampling using a relevant sampling frame was not possible. Thus, using data from INSEE (the French National Institute of Statistics), we performed quota sampling based on age and place of residence. Frequency matching was used to select the non-exposed subjects. Frequency matching involves the selection of an entire stratum of reference subjects (non-migrant groups) with matching-factor values equal to that of a stratum of index subjects (migrant group) [21]. The local-born French group was matched for age and socio-professional category. The non-migrant Tunisian group was matched for age and geographical origin (non-migrant Tunisians were born and are presently living in the same birthplace as the migrants who were surveyed).

The n = 150 sample size for each group was calculated to enable us to detect an odds ratio of 0.3 for migrants versus local-born French for diseases of the circulatory system based on a morbidity study [16], with an alpha-type error of 0.05 and a statistical power of 0.90.

### Data collection

Data was collected in 2004/2005 by interviewers who were bilingual in French and Tunisian. Interviewers were also trained and standardized for anthropometric and blood pressure measurements.

#### Health assessment

Blood pressure was measured twice using an automatic sphygmomanometer (Omron M5-I; Hoofddorp, Netherlands) validated by the French Health Security Agency. Hypertension was defined using WHO/ISH cut-offs [22]: grade-1 hypertension (systolic pressure ≥ 140 mm Hg and/or diastolic pressure ≥ 90 mm Hg) and grade-2 hypertension (systolic pressure ≥ 160 mm Hg and/or diastolic pressure ≥ 95 mm Hg). Hypertensive individuals also included people who declared they were receiving anti-hypertensive treatment.

All anthropometric measurements (standing height, weight and waist circumference) were measured twice according to standard procedures [23]. Overweight was defined according to the WHO classification as body mass index (BMI) ≥ 25 kg/m^2 ^[24]. Central obesity was defined according to WHO/ISH cut-offs points as waist circumference ≥ 94 cm [25].

Status with respect to NCDs (type-2 diabetes, cardiovascular diseases (CVD), and hypercholesterolemia) was evaluated from participants' answers to questions about specific health items in the questionnaire.

#### Assessment of socio-economic status and lifestyle

Educational status was divided into three categories: no schooling or primary school; secondary school, and university.

To assess economic status, correspondence analysis was performed on the matrix of indicator variables coding characteristics of dwelling, utilities and appliances. The score of each household on the first principal component was used as a summary index of household wealth [26] and the latter was introduced in analyses after breakdown into terciles of increasing economic level (low, medium and high).

The healthcare utilisation index was based on seven variables that reflected the use of the healthcare system. This summary index was built in a similar way to the economic index by correspondence analysis. It was also categorised in three levels: occasional, regular and frequent use.

To evaluate the physical activity level (PAL), we used a frequency questionnaire that assessed the time spent on different current activities: occupational habits, home activities, recreational activities, sports and travels to and from places during the last month, with specific attention to working days and holidays. Total daily physical activity (PA) (MET -h day^-1^) was estimated by summing the product of reported the time reported for each item by a MET value specific to each category of PA using a published Compendium of Physical Activities [27] and expressed as a daily average MET score (where MET is metabolic equivalent; 1 MET = 1 kcal/kg/hour). We estimated the Basal Metabolic Rate (BMR) from the weight, height and age of each person using the Henry equation [28]. We then calculated the total energy expenditure (TEE; Kcal ^day-1^) from BMR and total daily PA. The physical activity level (PAL) was assessed by PAL = TEE/BMR. The classification of lifestyles in relation to PAL was evaluated according to the FAO/WHO/UNU classification (sedentary lifestyle: 1.4–1.69; active lifestyle: 1.7–1.99; vigorous lifestyle: 2.0–2.4) [29].

The WHO-STEPS questionnaire was used to evaluate tobacco and alcohol consumption [30]. The variables used for the analysis were current smoking, and alcohol consumption during the last year. Subjects were considered as current smokers if they answered yes to the question "do you daily smoke cigarettes?" The variable relating to alcohol was based on the question "did you drink any alcohol during the last 12 months?", as this question appeared very discriminatory between groups.

To assess usual dietary intake during the past month, a validated quantitative Food Frequency Questionnaire [31] was adapted to the Tunisian context. After creation of a food composition table that was relevant for France and Tunisia, energy intake (kilojoules) was assessed using ESHA Food Processor software (version 8.3; ESHA Research Inc., Salem, OR, USA). The variable was then categorized into terciles of low, medium and high energy intake.

### Conceptual framework

**Figure 1:**
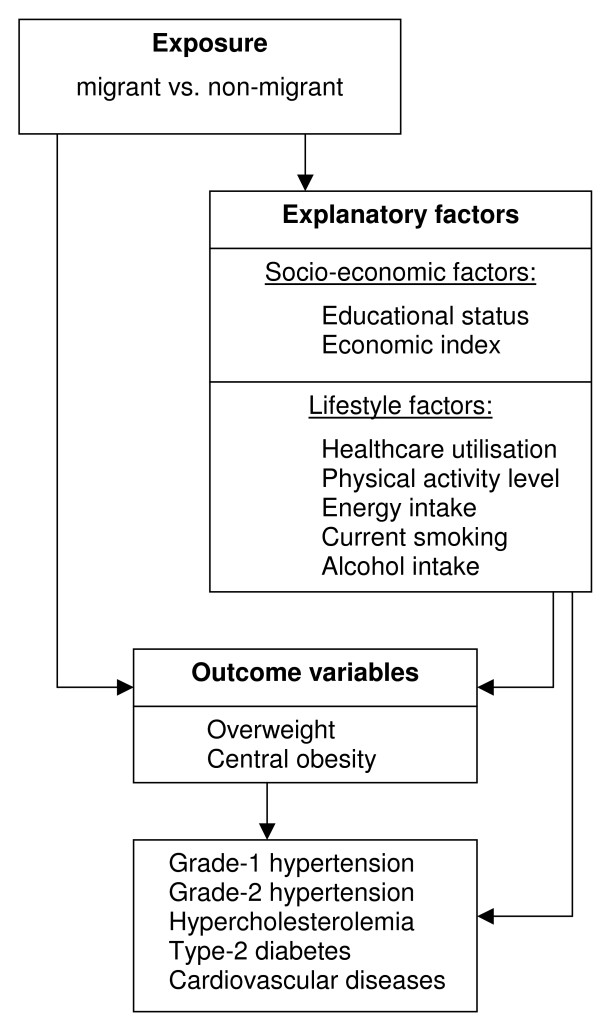
Conceptual framework.

To analyse the hypothesised relations between exposure, outcome variables and explanatory factors, the following hierarchical conceptual framework (Figure 1) [32] was used: migration could protect from overweight and NCDs through socio-economic factors, through lifestyle factors and through the overall context including the influence of overweight on NCDs, but also through factors that were not taken into consideration in this study (leftmost arrow) such as genetic factors, specific dietary characteristics and other factors which specifically affected each outcome variable.

### Statistical analysis

First, we compared overweight and morbidity in migrants and non-migrants. The strength of association was assessed by odds-ratios (OR) estimated by univariate conditional regression models to take frequency matching into account. Secondly, using embedded logistic regression models [33], we tested the potential mediators [34] of the effect of migration on the outcome variables significantly associated with migration status. The first model included only migration status. Next, the potential mediators were added sequentially, first the socio-economic variables, then the lifestyle variables, according to the conceptual framework. For hypertension, diabetes, cardiovascular diseases and hypercholesterolemia, overweight was also included as an explanatory variable.

At each step, the magnitude of the "mediating effect" was assessed by the change in the association between migration and the outcome variable (migration status OR) between thecurrent model and the previous model as measured by the mediating effect ratio (MER) defined as: (OR previous model – OR current model)/OR current model, similar to a confounding ratio [35]. We applied a quantitative criterion to see whether socio-economic and lifestyle variables had a mediating effect. To do so, a 20% reduction threshold was used to select mediating variables. The first type error rate was set at 0.05. Data entry and quality checks were performed using Epidata (version 3.1; Epidata Association, Odensen, Denmark), data management and statistical analyses were performed using SAS (version 9.1; SAS Institute, Cary, NC, USA).

### Ethical clearance

All interviewees gave their free written informed consent. This research complied with the principles of the Helsinki Declaration. The global protocol received approval from the French National Commission of Informatics and Freedom (CNIL) and from the Ministry of Health in Tunisia.

## Results

As three surveyed migrants subsequently withdrew their consent, the final total of subjects used in the analyses was 147.

### Socio-demographic and lifestyle characteristics

For migrants, the mean duration of residence was 23.0 ± 12.0 years (result not shown). The mean age of the migrants was 50.2 ± 13.2 and was not significantly different for the other groups due to the matching procedure used (Table 1).

**Table 1:** Socio-demographic and lifestyle characteristics of the different groups.

	Tunisian migrants		Local-born French		Tunisian migrants vs. local-born French	Tunisian migrants^1^		Tunisians		Tunisian migrants vs. Tunisians
				
	n	% or Mean (s.d.)	n	% or Mean (s.d.)	p-value	n	% or Mean (s.d.)	n	% or Mean (s.d.)	p-value
			
Age (years)	147	50.2 (13.2)	147	49.8 (13.9)	0.7	147	50.2 (13.2)	147	52.3 (16.3)	0.2
Educational level	147		147			147		147		
None or Primary		38.1%		16.3%			38.1%		42.2%	0.07
Secondary		42.2%		58.5%	0.0001		42.2%		40.8%	
University		19.7%		25.2%			19.7%		17.0%	
Economic level index	145		147			145		145		
Low		50.3%		16.3%	10^-4^		29.7%		38.6%	10^-4^
Medium		35.9%		32.0%			25.5%		40.7%	
High		13.8%		51.7%			44.8%		20.7%	
Healthcare utilisation level index	147		147			147		147		
Low		55.1%		11.6%			39.5%		24.5%	
Medium		23.1%		40.8%	10^-4^		36.0%		33.3%	0.002
High		21.8%		47.6%			25.5%		42.2%	
Current smoking	147	28.6%	147	34.7%	0.25	147	28.6%	147	40.8%	0.02
Alcohol consumption		19.1%		93.2%	<10^-4^		19.1%		12.9%	0.15
Physical activity level (3 groups)	147		147			147		147		
Sedentary or light active lifestyle		36.7%		35.4%			36.7%		55.1%	
Active or moderately active lifestyle		53.1%		43.5%	0.03		53.1%		32.0%	0.001
Vigorous or vigorously active lifestyle		10.2%		21.1%			10.2%		12.9%	
Categories of energy intake	145		147			145		147		
Low		29.6%		36.7%			35.2%		31.3%	
Medium		33.1%		34.0%	0.3		33.8%		33.3%	0.7
High		37.3%		29.2%			31.0%		35.4%	

1. Results relating to Tunisian migrants were repeated because the economic index, healthcare utilisation index and energy intake were built separately for the comparison of each group (migrants and French; migrants and Tunisians) and the breakdown into terciles did not give the same percentages for migrants in each case.

In comparison with the French, the migrants had a lower level of education, a lower economic level and lower healthcare utilisation level despite matching of the socio-professional category (Table 1). No differences were found with respect to current smoking and energy intake at the time of the survey. Prevalence of vigorous activity was lower among the migrants than among the French. Most French (93.2%) were alcohol consumers whereas this was the case of only 19.1% of migrants.

Results revealed no difference in educational level between migrants and non-migrants Tunisians. The migrants' economic level was higher but healthcare utilisation was lower than Tunisians. Prevalence of smoking and sedentary lifestyle was higher among Tunisians than migrants. There was no difference in alcohol consumption and energy intake (Table 1).

### Prevalence of overweight and of reported morbidity

Prevalence of overweight was lower among the migrants than among the French or among the Tunisians, but the difference was only significant in the comparison with the French (Table 2). There was no significant difference in central obesity: in each group 50% of the subjects were above the waist circumference cut-off value. Only 8.8% of migrants were hypertensive (grade-1) compared with 52.4% of the French and 42.9% of the Tunisians. There was no significant difference between the migrants and the French for declared diabetes or reported cardiovascular disease. The percentage of reported type-2 diabetes and of reported cardiovascular diseases in migrants was significantly lower than in Tunisians. The percentage of reported hypercholesterolemia was lower among the migrants than the French or the Tunisians (respectively: 2.7%; 19.0% and 11.6%).

**Table 2:** Prevalence of overweight and morbidity according to migration status.

	Tunisian migrants	Local-born French	Tunisians	Tunisian migrants vs. local-born French^1^	Tunisian migrants vs. Tunisians^1^
					
	%	%	%	p-value	p-value
				
Overweight (BMI >= 25 kg/mm^2^)	46.9	63.9	58.5	0.007	0.05
Central obesity (waist circumference >= 94 cm)	45.5	46.6	46.6	0.8	0.7
Grade 1 Hypertension	8.8	52.4	42.9	<10^-4^	<10^-4^
Grade 2 Hypertension	2.0	28.6	25.8	<10^-4^	<10^-4^
Hypercholesterolemia (declared)	2.7	19.0	11.6	0.0001	0.008
Diabetes (declared)	3.4	6.1	15.6	0.26	0.002
Cardiovascular diseases (declared)	3.4	6.0	8.8	0.1	0.03

1. Univariate conditional logistic regression model (n = 147 for each group)

### Effect of migration and mediating factors on overweight and morbidity

Detailed results of nested logistic regression models are given only for health outcomes for which significant mediating factors were found.

#### Comparison between migrants and French

After taking into account all the potential mediating variables the protective effect of migration status on overweight decreased from OR = 0.53 [0.33–0.84] to a no longer significant OR = 1.07 [0.55–2.54]. Results (Table 3) showed that the introduction of variables linked with the socio-economic context and subsequently, the introduction of individual factors such as healthcare utilisation, or physical activity level did not significantly alter the effect of migration status on overweight. On the contrary, adjusted for all other variables alcohol intake (MER = -48%) appears to be an important intermediate variable for the effect of migration status on overweight.

**Table 3:** Effect of migration status and of mediating factors on overweight (Tunisian migrants vs. local-born French).

Overweight (n = 143 for each group^1^)					
		Adjusted migration status effect			
	
Model number	Variables in models	Odds-Ratios	Confidence Interval	Mediating effect ratio^2^	p-value
	
**1**	**Migration status**	0.53	0.33–0.84		0.007
**2**	Migration status, **Educational status**	0.57	0.35–0.93	-7%	0.02
**3**	Migration status, Educational status, **Socio-economic index**	0.56	0.32–0.97	1%	0.04
**4**	Migration status, Educational status, Socio-economic index, **Healthcare utilisation index**	0.65	0.36–1.17	-13%	0.15
**5**	Migration status, Educational status, Socio-economic index, Healthcare utilisation index, **Physical activity level**	0.65	0.36–1.21	-1%	0.17
**6**	Migration status, Educational status, Socio-economic index, Healthcare utilisation index, Physical activity level, **Current smoking**	0.57	0.30–1.07	15%	0.08
**7**	Migration status, Educational status, Socio-economic index, Healthcare utilisation index, Physical activity level, Current smoking, **Energy intake**	0.56	0.30–1.05	2%	0.07
**8**	Migration status, Educational status, Socio-economic index, Healthcare utilisation index, Physical activity level, Current smoking, Energy intake, **Alcohol intake**	1.07	0.45–2.54	-48%	0.87

1. Embedded conditional logistic regression models including only men with no missing value for all variables

2. Mediating effect ratio was defined as: (OR previous model – OR current model)/OR current model

Regarding hypertension (grade-1 and grade-2) and hypercholesterolemia, the results of the first model showed a strong protective effect of being a migrant vs. being French. The odd-ratios were respectively 0.06 [0.03–0.14], 0.04 [0.01–0.15] and 0.11 [0.04–0.34]. The migration status odd-ratios for the more complete models were still respectively 0.08 [0.02–0.33], 0.02 [0.01–0.27] and 0.03 [0.01–0.45] indicating that there was no straightforward mediating effect (results not shown).

#### Comparison between migrants and Tunisians

Regarding type-2 diabetes, the result of the first model (Table 4) showed a strong protective effect of migration status which appears to be partly mediated through individual factors such as healthcare utilisation, physical activity level and current smoking. The mediating effect of overweight was borderline. It should be noted that the introduction of the educational level in the model led to an increase in the association (Table 4).

**Table 4:** Effect of migration status and of mediating factors on diabetes (Tunisian migrants vs. Tunisians).

Diabetes (n = 141 for each group^1^)					
		Adjusted migration status effect			
	
Model number	Variables in models	Odds-Ratios	Confidence Interval	Mediating effect ratio^2^	p-values

**1**	**Migration status**	0.18	0.06–0.54		0.002
**2**	Migration status,**Educational status**	0.14	0.04–0.45	29%	0.001
**3**	Migration status, Educational status, **Socio-economic index**	0.13	0.04–0.47	8%	0.002
**4**	Migration status, Educational status, Socio-economic index, **Healthcare utilisation index**	0.21	0.06–0.78	-38%	0.02
**5**	Migration status, Educational status, Socio-economic index, Healthcare utilisation index, **Physical activity level**	0.29	0.08–1.07	-28%	0.06
**6**	Migration status, Educational status, Socio-economic index, Healthcare utilisation index, Physical activity level, **Current smoking**	0.38	0.10–1.41	-24%	0.15
**7**	Migration status, Educational status, Socio-economic index, Healthcare utilisation index, Physical activity level, Current smoking, **Energy intake**	0.37	0.10–1.42	3%	0.15
**8**	Migration status, Educational status, Socio-economic index, Healthcare utilisation index, Physical activity level, Current smoking, Energy intake, **Alcohol intake**	0.34	0.08–1.39	9%	0.13
**9**	Migration status, Educational status, Socio-economic index, Healthcare utilisation index, Physical activity level, Current smoking, Energy intake, Alcohol intake, **Overweight**	0.42	0.10–1.84	-19%	0.25

1. Embedded conditional logistic regression models including only men with no missing value for all variables

2. Mediating effect ratio was defined as: (OR previous model – OR current model)/OR current model

Migration status had a protective effect on cardiovascular diseases (Table 5) and, given the same hierarchical approach, its effect appears to be mediated by individual factors such as healthcare utilisation and energy intake.

**Table 5:** Effect of the migration status and of mediating factors on cardiovascular diseases (Tunisian migrants vs. Tunisians).

Cardiovascular diseases (n = 141 for each group^1^)					
		Adjusted migration status effect			
	
Model number	Variables in models	Odds-Ratios	Confidence Interval	Mediating effect ratio^2^	p-value
	
**1**	**Migration status**	0.25	0.07–0.88		0.03
**2**	Migration status, **Educational status**	0.23	0.06–0.86	9%	0.03
**3**	Migration status, Educational status, **Socio-economic index**	0.28	0.06–1.26	-18%	0.09
**4**	Migration status, Educational status, Socio-economic index, **Healthcare utilisation index**	0.48	0.08–2.69	-42%	0.40
**5**	Migration status, Educational status, Socio-economic index, Healthcare utilisation index, **Physical activity level**	0.50	0.08–3.23	-4%	0.46
**6**	Migration status, Educational status, Socio-economic index, Healthcare utilisation index, Physical activity level , **Current smoking**	0.46	0.06–3.29	9%	0.44
**7**	Migration status, Educational status, Socio-economic index, Healthcare utilisation index, Physical activity level, Current smoking, **Energy intake**	0.69	0.09–5.64	-33%	0.73
**8**	Migration status, Educational status, Socio-economic index, Healthcare utilisation index, Physical activity level, Current smoking, Energy intake, **Alcohol intake**	0.69	0.09–5.61	0%	0.73
**9**	Migration status, Educational status, Socio-economic index, Healthcare utilisation index, Physical activity level, Current smoking, Energy intake, Alcohol intake, **Overweight**	0.71	0.08–6.48	-3%	0.76

1. Embedded conditional logistic regression models including only men with no missing value for all variables

2. Mediating effect ratio was defined as: (OR previous model – OR current model)/OR current model.

Finally, our results showed a protective effect of migration status on grade-1 hypertension (OR = 0.07 [0.03–0.18]), grade-2 hypertension (OR = 0.06 [0.02–0.20]) and hypercholesterolemia (OR = 0.23 [0.08–0.63]) but even in the more complete models, for these three outcome variables the association with migration status remained more or less unchanged (results not shown).

## Discussion

The aim of this study was to assess the effect of migration on overweight and morbidity linked to some NCDs among Tunisian immigrants presently residing in the south of France and check whether any factors could explain this effect. Compared to local-born populations, Tunisian migrants exhibited a quite different morbidity pattern with a lower prevalence of overweight, reported hypercholesterolemia and hypertension. Thus our results appear to be in line with the existence of a Mediterranean migrant paradox in France [17].

In view of their low socioeconomic status and poor working conditions, higher mortality and morbidity among migrants would be expected. Indeed, studies in free health centres, which attract the most underprivileged migrants, showed that the frequencies of psychiatric disorders and communicable diseases such as infections by HIV and hepatitis C virus, parasitism and tuberculosis are particularly high among migrants [36]. This is particularly the case among Sub-Saharan African migrants but not among North African migrants. Indeed, there is also evidence of health benefits among migrants originating from North Africa countries, mainly for diseases of affluence but not solely [16].

In order to evaluate the extent of the change in health risk after migration, we analysed data of matched non-migrant Tunisians from the same geographical origin. This study suggests that migrants are also better protected from some NCDs (hypertension, hypercholesterolemia, type-2 diabetes and cardiovascular diseases) than their counterparts in Tunisia. These results are particularly interesting because they are in concordance with the shift in obesity and the attendant risks of NCDs in Tunisia over recent decades. Indeed, Tunisia is undergoing a rapid nutritional transition in the context of economic development [18] and there has been an almost twofold increase in the prevalence of obesity and in the associated risks of NCDs in the last seven years [37].

### Comparison with local-born French

Here the main result was the predominant mediating effect of alcohol intake on overweight. Detailed data showed that mean daily alcohol consumption was 0.6 ± 0.1 drinks for the Tunisian migrants compared with 2.6 ± 0.1 drinks for the French. Data from a French national survey also showed that North African migrants have lower alcohol consumption [38] than local-born French, together with a lower risk of mortality from upper aero-digestive and liver cancers [14]. It appears that the observance of the religious prohibition on alcohol consumption by a high percentage of Tunisian migrants has indeed a protective effect on overweight. According to Jequier [39], the relation between alcohol consumption and body weight remains an enigma for nutritionists. As a matter of fact, epidemiologic evidence is inconsistent with numerous studies suggesting absent or only weak positive relations in men [40]. However, alcohol is the second most energy dense macronutrient consumed [41] and is known to reduce oxidation of fat and to favour fat storage, which may result in weight gain [40]. In this way, our results support the conclusion that regular high alcohol intake could contribute to overweight, while abstainers could be protected.

The association of migration with hypertension and with hypercholesterolemia remained mostly unchanged even in the more complete models. It seems that other determinants that were not included in this study, such as dietary characteristics, could have an effect on hypertension and hypercholesterolemia. Indeed, a separate publication revealed that the conservation of a healthier diet through better diet adequacy was sufficient to provide some protection from hypertension and hypercholesterolemia among migrants [42]. Moreover, the fact they consumed more fibre than the local-born French also explained the favourable association between migration status and hypertension. Our findings on migrants also suggest that their higher intake of vitamin C, and particularly of fresh fruits as sources of vitamin C, could partly protect them from the onset of hypercholesterolemia compared to local-born French.

### Comparison with non-migrant Tunisians

Given that the migrants use less healthcare than non-migrant Tunisians, healthcare use cannot be interpreted as a mediator of the protective effect of migration. Alternatively, this may reflect a reverse causal relationship where the outcome (diabetes) is the causal factor of the hypothesised mediator (healthcare utilisation). Indeed, frequent use of health care may result from the onset of type-2 diabetes, rather than represent a preventive effect on type-2 diabetes. Another explanation may be underestimation of the prevalence of type-2 diabetes among the migrants because of non-diagnosed diseases, given their lower use of healthcare.

A number of prospective studies showed that higher levels of physical activity reduced the risk of type-2 diabetes [43] and the beneficial effects of physical activity on the incidence of diabetes appear to be mainly due to the effect of muscular activity on insulin sensitivity [44]. However, a preventive effect of physical activity on type-2 diabetes through reducing BMI cannot be excluded [45]. Moreover, our data showed that overweight influenced the protective effect of migration. It thus appears plausible that the active lifestyle, which is more prevalent among the migrants than among non-migrant Tunisians, and its effect on overweight, protect the migrants from type-2 diabetes.

There is considerable epidemiological evidence linking smoking with insulin resistance. Consistent results from both cross-sectional and prospective studies show that smoking increases the chance of developing type-2 diabetes [46]. Smoking appears to be less prevalent among the migrants than among their counterparts in Tunisia and this may thus partly explain the protective effect of migration on type-2 diabetes. The difference in current smoking may be due to regular preventive actions against smoking implemented in France including taxes that make tobacco more expensive in France than in Tunisia with respect to the standard of living.

Considering healthcare utilisation as a mediator of the migration effect on CVD, we can also hypothesize reverse causality or underestimation of the prevalence of CVD among migrants as described above for type-2 diabetes.

Mean energy intake appears to have a mediating effect on CVD. One possible explanation is the lower consumption of sugar among migrants than among Tunisians [42]. Indeed, higher intakes of high energy dense foods, such as sugar, are considered to be the primary exposures that lead to increased total caloric intake, which in turn causes diseases [47]. Thus, the Tunisian migrants in France eat healthier diets (low consumption of high energy dense foods) associated with a preventive effect for CVD.

As was the case for the French, other mediating factors may also play an important role in the associations between migration status and hypertension and hypercholesterolemia. Indeed, the conceptual framework used in this study did not take into account all the determinants of overweight and NCDs. Detailed analyses of the precise influence of the dietary characteristics of migrants on hypertension and hypercholesterolemia did not also reveal any dietary mediating factors [42]. A possible explanation of the health advantages of Tunisian migrants compared to the non-migrant population of their home country could be the "healthy migrant effect", a selection bias in relation with migration. Indeed, Tunisian migrants (North African migrants) in France have undergone a considerable selection process. Darmon and Khlat [17] reported that migrants are among the healthiest of their country of origin, as they are subjected to positive selection, either via self-selection or selection due to mandatory health controls on entrance to the host country.

The characteristics of the study should of course be taken into account when the results are interpreted. Concerning selection bias, although the non-random nature of the sample may be an issue, the quota sampling strategy was the only one possible to study Tunisian migrant populations in France. In addition, as the survey was limited to the south of France and the sample was small, generalisation might be problematic. However, the statistical power appears to be sufficient with regard to the objectives of this study. Also, the maximum number of parameters for the more complex models is well under the 10% threshold usually recommended for the ratio of number of parameters to number of observations [48]. Another feature of the study was the use of reported data concerning morbidity (except for overweight and hypertension). In a context with large provision of health care, easy access to healthcare and widely circulated sanitary information, diagnosed morbidity and reported morbidity could overlap to a considerable extent [49]. But, in France, despite a favourable context, inequality of healthcare utilisation still exists. Most studies show that migrants use less healthcare than their French peers [36]. This difference could influence the comparison of reported morbidity.

## Conclusion

These findings do provide evidence that Tunisian migrant men residing in France enjoy better health with respect to overweight and NCDs than local-born French. Our study thus supports works that brought to light a Mediterranean migrant paradox in France on mortality [14,15] and morbidity [16]. In addition, results of the original comparison between migrants and their non-migrant counterparts in Tunisia suggest that not only cultural (alcohol consumption) but also environmental factors (physical activity, smoking habits) are the main factors involved in the paradox.

These factors partially explain the relatively better health of Tunisian migrant men in France, whereas the favourable effect of migration on hypertension and hypercholesterolemia compared with local-born French could be explained by the conservation of some dietary characteristics [42]. This result is in line with Powles' hypothesis concerning the migrant paradox [13]: the continuation of traditional behaviours favourable to health and better access to effective medical care could protect migrants, particularly from obesity and NCDs.

Finally, it should be noted that these results may be specific to migrant men. North African women are subject to different conditions of life and do not present the same health advantage as men either in the host country [16] or in their country of origin [19]. Consequently, it cannot be inferred that the same conclusions apply to them. A specific study relating to the health status of North African migrant women would be required to investigate their situation.

Better knowledge of health and healthy lifestyles of migrants will facilitate the development of culturally appropriate preventive actions. Migrants represent a large permanent minority and it is essential to assess their health status and to understand its specific determinants. Further work is planned to explore in more detail differences in explanatory variables, particularly healthy behaviours, to provide a better insight into migration-health relationships.

## Competing interests

The author(s) declare that they have no competing interests.

## Authors' contributions

CM designed the study, carried out the surveys, performed the statistical analysis and drafted the manuscript. PT supervised the statistical analysis and participated in drafting the manuscript. SED performed data management and statistical analyses. JEA helped to coordinate the survey in Tunisia and to draft the manuscript. FD was involved in drafting the manuscript and gave his expert comments and suggestions to improve it. BM was involved in the conception and design of the study, in the interpretation of data and helped draft the manuscript.

All authors have read and approved the final manuscript.

## Pre-publication history

The pre-publication history for this paper can be accessed here:


